# Violence against housemaids in an Ethiopian town during the early phase of the COVID-19 pandemic: a cross-sectional study

**DOI:** 10.1186/s12905-023-02530-w

**Published:** 2023-09-12

**Authors:** Metadel Adane, Helmut Kloos, Yordanos Mezemir, Amare Muche, Erkihun Amsalu

**Affiliations:** 1 Department of Environmental Health, College of Medicine and Health Sciences, Wollo University, Kombolcha, Ethiopia; 2 Department of Epidemiology and Biostatistics, University of California, San Francisco, USA; 3 Debre Birhan Health Science College, Debre Birhan, Ethiopia; 4 Department of Epidemiology and Biostatistics, School of Public Health, College of Medicine Health Sciences, Wollo University, Kombolcha, Ethiopia

**Keywords:** COVID-19 pandemic, Housemaids, Sexual violence, Physical violence, Kombolcha Town, Ethiopia

## Abstract

**Background:**

Violence against women is a global public health problem that has numerous adverse effects. However, published literature regarding violence against housemaids during the COVID-19 pandemic in Ethiopia is lacking. The current study aims to explore the experiences of violence and associated factors among housemaids in Ethiopia. The findings may be useful to the design appropriate policies, programs and strategies to reduce the problem.

**Methods:**

A community-based cross-sectional study was conducted from January to March, 2021 in Kombolcha Town, Ethiopia. A total of 215 housemaids aged 14 years and older were included in the study using a simple random sampling technique. A multivariable logistic regression model with 95% CI (confidence interval) was applied to identify significant factors of physical and sexual violence. Variables with a *P*-value < 0.05 were declared as factors significantly associated with violence.

**Results:**

Among 215 housemaids, 33.49% (95% CI: 27.13–39.85%) reported physical violence and 21.4% (95% CI: 15.87–26.92) reported sexual violence during the COVID-19 pandemic. Thus, housemaids aged 19–23 years (AOR = 2.64, 95% CI: 1.01–6.89), who had a male employer (AOR = 2.39, 95% CI: 1.05–5.45), whose employers chewed *chat* (*Catha edulis*) (AOR = 3.78, 95% CI: 1.73–8.29), or drank alcohol (AOR = 2.90, 95% CI: 1.17–7.17) experienced more physical violence. Sexual violence was also associated with employers’ alcohol consumption (AOR = 9.72, 95% CI: 3.12–20.31), employers’ *chat* chewing (AOR = 7.40, 95% CI: 2.26–14.21) and male employers (AOR = 3.23, 95% CI: 1.22–8.52).

**Conclusion:**

The findings indicate that one in five housemaids and one in three housemaids experienced sexual violence and physical violence, respectively. Housemaids aged 19–23 years, having a male employer, having an employer who chewed *chat* (*Catha edulis*) or who drank alcohol were factors associated with physical violence, whereas employers’ alcohol consumption, employers’ *chat* chewing and male employers were factors associated with sexual violence.

**Supplementary Information:**

The online version contains supplementary material available at 10.1186/s12905-023-02530-w.

## Background

Violence against women is a public health problem as well as a basic violation of women’s human rights that often results in injury, death, and sexual and psychological harm [1, 2]. Purposeful use of physical force is considered “physical violence,” while forcing a woman to engage in a sexual act is referred to as “sexual violence” [3].

Worldwide, an estimated one in three women has been physically or sexually abused; and one in five experience rape or attempted rape in their lifetime, but this are probably underestimates [1]. Violence against women is one of the main contributors to poor sexual and reproductive health, leading to unintended pregnancy, self-induced abortions, gynecological problems, sexual dysfunction and sexually transmitted infections (STIs), including HIV [4, 5].

Studies have reported that socioeconomic marginalization, poor mental health, alcohol misuse by a partner, unplanned pregnancies, young age and a history of childhood abuse are risk factors for violence against housemaids [6–10]. Globally, data show that reports of domestic violence have increased during the COVID-19 pandemic [11, 12]. In China, housemaid violence tripled during the mandatory stay-at-home period [13]. Violence increased also in the UK, Canada, USA, Italy, Spain, and Australia during the COVID-19 pandemic [11, 14]. In Kenya, Somalia, South Africa, Niger, Tunisia, Zimbabwe, and Ethiopia, housemaid violence has been reported as high as before the pandemic [15].

In Ethiopia, data from several hospitals in Addis Ababa showed that between mid-March and mid-May 2020 more than 100 girls were raped, some of them by close family members [16].

Socioeconomic instability related to stay-at-home orders and business closures increase substance abuse and lack of community support, all of which contribute to violence [17]. Women in Ethiopia and other developing countries face numerous barriers to accessing justice for gender-based violence and rarely report violence committed against them due to low conviction rates, disruption of court processes during the COVID-19 pandemic, and cultural factors, factors which shield perpetrators [18].

In Ethiopia, seasonal and long-term rural–urban migration for domestic work is highly prevalent. The main destinations of these migrants are regional and zonal towns, which provide migrants with opportunities for domestic work as housemaids [19]. Domestic work is one of the least protected sectors under the labor law and poor monitoring and implementation of the existing laws puts housemaids in a highly disadvantaged position, exacerbating their vulnerability to sexual abuse and violence [20, 21].

Housemaids are highly vulnerable to violence due to the nature of their work, socio-economic exclusion, and residence in the homes of their employers. But published literature on violence against housemaids during the COVID-19 pandemic in Ethiopia is limited. To fill this gap the current study aims to explore the experiences and risk factors of physical and sexual violence among housemaids aged 14 years and older in Kombolcha Town during the COVID-19 pandemic. The findings may be useful in the design of appropriate policies, programs and strategies that address the vulnerabilities of housemaids and reduce sexual and physical violence among housemaids. In addition, this study also provides useful information to labor and social affairs and health professionals, as well as policy makers planning and implementing prevention programs.

## Methods and materials

### Study setting, design and period

A community based cross-sectional design was utilized from January to March 2021 to assess violence among housemaids aged 14 years and older during the COVID-19 pandemic. The study was carried out in Kombolcha Town in northeast Ethiopia. Kombolcha is located in South Wollo Zone in Amhara Region at 1,800 m altitude, latitude 11°5′N and longitude of 39°44′E.

Data obtained from the South Wollo Zone Administration show that the population of Dessie Town was 102,350 in 2020. The great majority of the rural zonal population practices subsistence agriculture focused on grain cultivation and livestock rearing. South Wollo Zone is subject to frequent droughts and famines that cause many people, including young females, to move to urban areas, including Kombolcha Town, to seek work. Kombolcha Town is divided into 4 urban *kebeles* (the smallest administrative unit in Ethiopia) containing 28,040 households and a total of 3,467 housemaids aged 14 years and older in 2021, according to the Kombolcha Town Administration Office.

### Source and study population

All housemaids aged 14 years and older in Kombolcha Town were the source population and all selected housemaids aged 14 years and older living in the town during data collection were considered as the study population. Housemaids who were critically ill and those who were unable to communicate were excluded from the study.

### Sample size determination and sampling technique

The sample size was determined using a single population proportion formula:

$$\mathrm n\;=\;\frac{\left({\mathrm{Za}}_{/2}\right)^2\;\mathrm P\left(1-\mathrm P\right)}{\mathrm d^2}$$

Taking 16.3% prevalence of physical violence against housemaids from a study conducted in Mekele City in northern Ethiopia [22], with an assumed 95% CI and 5% margin of error, giving a sample size of 204. By adding 10% non-response rate, the total sample size was 224. For the second objective, considering two independent variables, a double population proportion formula was adopted from previous studies [22, 23]. Then, the sample size was computed using Epi-Info version 7.0 software and found to be 141. Thus, the maximum adequate sample size used for this study was 224.

According to the Kombolcha Town Administration, there are 4 *kebeles* with a total of 3,467 housemaids aged 14 years and above in January 2021. The sample size was proportionally allocated for each *kebele* based on the number of housemaids in the respective *kebeles.* Then a simple random sampling technique using the lottery method was employed to select study participants from the Kombolcha Town Administration files. Households of housemaid aged 14 years or above were coded to properly identify them. The data collectors started at the bench mark of the known location and then walked straight forward to identify each house. When more than one housemaid was present in a selected household, a lottery method was used to select one of them. When the housemaid in the selected household was not available, that house was revisited the same day. If again not available, the household was revisited a third time and considered as non-respondent if still absent.

### Study variables

The dependent variables of the study were physical violence and sexual violence among housemaids. Independent variables were socio-demographic characteristics of housemaids (age, previous residence, marital status, educational status, religion, monthly income, work experience, type of agreement with the employer, reasons for being a housemaid), housemaid’s current family characteristics (parents live together, father’s education, mother’s education), housemaid’s behavioral characteristics (chewing *chat*, frequency of *chat* chewing, smoking, drinking alcohol, frequency of drinking alcohol, using cocaine, using *shisha*, using marijuana), and employer characteristics (education, gender, occupation, chewing *chat*, drinking alcohol, frequency of chewing *chat*, and frequency of drinking alcohol).

### Operational definition

#### Housemaid

refers to a female domestic worker employed to do housework with or without contract and either living or not living in the employer’s home [24, 25].

#### Physical violence

was defined as any act of slapping a housemaid or throwing something at her, pushing or shoving her or pulling her hair, kicking or dragging her, beating her, punching her with a fist or with something else that could hurt her, choking or burning and threatening her with a weapon [26, 27].

#### Sexual violence

was defined as any acts of forced sexual intercourse or in this study, we also consider sexual violence as unwanted touching of breasts or the genital area, making sexually explicit remarks and threats of rape [26, 27].

### Data collection tool and techniques

For data collection, interviewers administered a questionnaire adopted from validated WHO multi-country study on violence against women [26] (Additional file 1). The questionnaire was originally prepared in English and then translated into Amharic (local language) and back into English to ensure its consistency and accuracy. Four MPH supervisors and 8 unmarried female nurses with BSc degrees and well-experienced in data collection were recruited for data collection and trained for 2 days about the objectives and relevance of the study, confidentiality of information, the respondents’ rights and interviewing techniques.

The questionnaire was pretested on 5% of the total sample size in Dessie Town before the actual data collection to assure the validity of the questionnaire. Data were collected during face-to-face interviews with each respondent in a private and secure place, usually in a room or other space without the presence of the employer.

### Data management and analysis

Data were entered into Epi-Info version 7 and exported to STATA version 14 software for further analysis. Continuous variables were summarized using mean and standard deviation. Bi-variable and multivariable logistic regression analysis was done to determine associations between independent and dependent variables. Variables having *P*-value < 0.25 in the bivariate analysis were selected using forward procedures and examined in multivariable logistic regression analysis to control possible confounders as described by Hosmer and Lemeshow [28].

Adjusted odds ratio (AOR) with 95% confidence interval (CI) was used to measure strength and direction of associations. Thus, variables having *P*-values less than 0.05 in the final model were considered as factors significantly associated with violence. Multi-collinearity between variables was checked using a standard error of the coefficient with cut-off point 2.0. The Hosmer and Lemeshow goodness-of-fit test (28) was used to determine the model fitness of the adjusted multivariable analysis with cut-off-point *p*-value > 0.05, which was found to be 0.89.

## Results

### Characteristics of housemaids

Of the 224 housemaids included in the study, 215 fully responded the questionnaire, giving a response rate of 95.98%. The mean age of respondents was 20.66 years, with SD (standard deviation) ± 3.68, while half were 19–23 years old. Ninety-three (43.25%) of the housemaids had attended primary school, 170 (79.07%) were currently unmarried and the mean of their monthly salary was 978.28 (SD ± 198.97) Ethiopian Birr (Table 1).

**Table 1:** Characteristics of housemaids in Kombolcha town, northeast Ethiopia, January to March, 2021 (*N* = 215)

Variable	Category	n (%)
Age (years)	14–18	66(30.70)
	19–23	109(50.70)
	24–35	40 (18.60)
Religion	Christian	121(56.28)
	Muslim	94(43.72)
Marital status	Unmarried	170(79.07)
	Married	45(20.93)
Education status	Unable to read and write	20(9.30)
	No formal education	25(11.63)
	Primary school	93 (43.25)
	Secondary school	66 (30.70)
	University	11(5.12)
Monthly salary (Ethiopian Birr)^a^	< 978.28	83 (38.60)
	≥ 978.28	132 (61.40)
Previous residence	Rural	104 (48.37)
	Urban	111 (51.63)
Work experience	< 1 year	119 (55.35)
	1–3 years	72 (33.49)
	> 3 years	24 (11.16)
Type of agreement with employer	Temporary written contract	19 (8.84)
	Daily	26 (12.09)
	Permanent written contract	170 (79.07)
With whom do you live?	With employer	182 (84.65)
	With my family	18 (8.37)
	Other^b^	15 (6.98)
Reason for being a housemaid	Lack of employment opportunities	154 (71.63)
	Divorced	30 (13.95)
	Opposing early marriage	31 (14.42)

^a^1 USD **=** 36.94 Ethiopia Birr (ETB) during the study period

Other^b^: Housemaids who live alone, live with a female friend, or live with their husband

More than half (111, 51.63%) of the housemaids had grown up in urban areas and 119 (55.35%) of them had worked for their current employer for less than one year. Regarding the type of employment agreement with the employer, 170 (79.07%) of the housemaids had a contract with their employer and 182 (84.65%) were living with their employer. Most of the housemaids 154 (71.63%) chose to work as a housemaid due to lack of other employment opportunities (Table 1).

### Housemaids’ family characteristics

Housemaids were asked about their family history. More than half (134, 62.33%), of their parents were living together. Nearly two-fifths of them were unable to read and write, 93 (43.25%) had completed primary school and 13 (6.05%) secondary school or above (Table 1). Fewer than 8% of their fathers and mothers had completed secondary school or above. More than four-fifths 175 (81.40%) of the housemaids considered their monthly salaries to be insufficient (Table 2).

**Table 2:** Housemaids current family characteristics in Kombolcha town, Ethiopia, January to March, 2021 (*N* = 215)

Variable	Category	Frequency (n)	Percentage (%)
Parents live together	Yes	134	62.33
	No	81	37. 67
Father’s education	Unable to read and write	84	39.07
	No formal education	73	33.95
	Primary education	45	20.93
	Secondary or above	13	6.05
Mother’s education	Unable to read and write	85	39.53
	No formal education	78	36.28
	Primary education	35	16.28
	Secondary or above	17	7.91

### Housemaids’ substance use

Forty-two (19.53%) of the housemaids drank alcohol, nearly all (41, 97.62%) consuming alcohol sometimes. Sixty-one (28.37%) of them chewed *chat, (Catha edulis)*, 54(88.52%) chewing *chat* sometimes. Thirty-one (14.42%) of the housemaids smoked using water pipes (*shisha*). None of the housemaids said yes to use of cocaine or marijuana (Table 3).

**Table 3:** Substance use-related behaviors of housemaids in Kombolcha town, Ethiopia, January to March, 2021 (*N* = 215)

Variable	Category	Frequency (n)	Percentage (%)
Do you drink alcohol?	Yes	42	19.53
	No	173	80.47
Frequency of drinking alcohol (*n* = 42)	Regularly	1	2.38
	Sometimes	41	97.62
Do you chew *chat*?	Yes	61	28.37
	No	154	71.63
Frequency of chewing *chat* (*n* = 61)	Regularly	7	11.48
	Sometimes	54	88.52
Do you use *shisha*?	Yes	31	14.42
	No	184	85.58

### Current employers characteristics

Nearly half (101, 46.98%) of the current employers had a diploma or university education. Half of the current employers (108, 50.23%) were government employees and more than half (112, 52.09%) of them were *chat* chewers, 58 (51.79%) of them chewing once or twice a week and 17.79% daily. Sixty-three (29.30%) of the current employers used alcohol, of whom 38 (61.29%) of them drank once or twice a week (Table 4).

**Table 4:** Characteristics of current employers of housemaids in Kombolcha Town, Ethiopia, January to March, 2021 (*N* = 215)

Variable	Category	Frequency (n)	Percentage (%)
Education of current employer	Primary school	18	8.37
	Secondary school	45	20.93
	Diploma or above	101	46.98
	Don’t know	51	23.72
Gender of current employer	Male	67	31.16
	Female	42	19.53
	Both male and female	106	49.30
Occupation of current employer	Merchant	52	24.19
	Government employee	108	50.23
	Private worker	55	25.58
Does current employer you chew *chat*?	Yes	112	52.09
	No	103	47.91
Frequency of chewing *chat* of current employer (*n* = 112)	Every day	20	17.86
	Once or twice a week	58	51.79
	1–3 times a month	24	21.43
	Occasionally	10	8.93
Does current employer you drink alcohol?	Yes	63	29.30
	No	152	70.70
Frequency of drinking alcohol of current employer (*n* = 63)	Every day	5	8.06
	Once or twice a week	38	61.29
	1–3 times a month	13	20.97
	Less than once a month	6	9.68

### Experiences of physical violence

Of the 215 respondents, 72 (33.49%) had experienced physical violence (95% CI: 27.13, 39.85%) at their present or previous jobs. Among these, 33 (45.84%) had experienced more physical violence since the lockdown for the COVID-19 pandemic. Regarding the kind of physical violence experienced, 26 (36.11%) of the housemaids had been slapped or punched with a fist, 33 (45.83%) of them had been hit with an object, and 13 (18.06%) reported being kicked. About one-third (35, 48.61%) of the housemaids had not reported the violence committed against them (Table 5**)**.

**Table 5:** Experiences and impacts of physical violence among housemaids in Kombolcha, Ethiopia, January to March, 2021 (*N* = 215)

Variable^a^	Category	Frequency	Percentage (%)
Have you experienced physical violence?	Yes	72	33.49
	No	143	66.51
Type of physical violence (*n* = 72)	Slapped or punched with a fist	26	36.11
	Hit with an object	33	45.83
	Kicked	13	18.06
Have you experienced physical violence since lockdown for COVID-19? ((*n* = 72)	Yes	33	45.84
	No	39	54.16
Impact of physical violence (*n* = 72)	Sustained injuries	10	13.89
	Became depressed	49	68.06
	Became fearful	4	5.56
	No major impact	9	12.50
Was violence justified because of your behavior or attitude? (*n* = 72)	Yes	19	26.39
	No	53	73.61
Did you report physical violence? (*n* = 72)	No	35	48.61
	I talked to my close friends	28	38.89
	I left my job	9	12.50
Is violence normal and tolerable? (*n* = 72)	Yes	18	25.00
	No	54	75.00
Have you considered changing jobs due to violence? (*n* = 72)	Yes	50	69.44
	No	22	30.56
Do you want to change your occupation? (*n* = 68)	Yes	39	57.35
	No	29	42.65

^a^Impacts of physical violence reporting on the subset of women who have experienced violence

The study also showed that 53 (73.61%) of the housemaids considered the violence not to have been justified by their behavior or attitude. More than two-thirds 49 (68.06%) of the respondents experienced depression due to violence, 50 (69.44%) considered changing jobs due to violence and 39 (57.35%) of them wanted to change their occupation (Table 5).

### Factors associated with physical violence

Age, religion, monthly income, previous residence, work experience, marital status, use of alcohol and *shisha* by housemaids, and gender of and alcohol and *chat* use by employer were significant variables in the bi-variable analysis at *P*-values < 0.25. They were entered into a multivariable model to control for possible confounding and to identify significant predicators. In the multivariate logistic regression analysis, age of housemaids, gender of employer, and employers chewing *chat* or drinking alcohol were statistically significant factors of physical violence among housemaids.

The odds of physical violence were nearly three times higher against those aged 19–23 years than those aged 14–18 years (AOR = 2.64, 95% CI: 1.01–6.89). Housemaids who had a male employer were 2.4 times more likely (AOR = 2.39, 95% CI:1.05–5.45) to experience physical violence than housemaids who had both male and female employers. Housemaids whose employer chewed *chat* were 3.8 times more likely (AOR = 3.78, 95% CI:1.73–8.29) to have experienced physical violence than their counterparts whose employers did not chew *chat*. Similarly, housemaids whose employer used alcohol were 2.9 times more likely (AOR = 2.90, 95% CI:1.17–7.17) to have experienced physical violence than their counterparts whose employers were non-drinkers (Table 6).

**Table 6:** Factors associated with physical violence against housemaids in Kombolcha Town, Ethiopia, January to March, 2021 (*N* = 215)

Variable	Category	Physical violence		COR(95%CI)	AOR(95%CI)	*p*-value
		Yes	No			
Age of housemaid	14–18	10	56	1.00	1.00	
	19–23	44	65	3.79(1.74, 8.22)	**2.64(1.01, 6.89)** ^a^	**0.047**
	24–35	18	22	4.58(1.83,11.46)	2.78(0.82, 9.40)	0.100
Religion of housemaid	Christian	54	67	1.00	1.00	
	Muslim	18	76	0.29(0.15, 0.54)	0.53(0.22, 1.23)	0.141
Monthly salary (Ethiopia Birr)	< 978.28	21	62	1.00	1.00	
	≥ 978.28	51	81	1.86(1.01, 3.41)	1.29(0.59, 2.80)	0.512
Previous residence of housemaid	Rural	40	64	1.54 (0.87, 2.72)	1.55(1.25, 2.29	0.147
	Urban	32	79	1.00	1.00	
Duration of work as housemaid	< 1 year	25	94	0.26(0.10, 0.66)	0.41(0.12, 1.39)	0.153
	1–3 years	35	37	0.94(0.37, 2.38)	0.90(0.26, 3.01)	0.865
	> 3 years	12	12	1.00	1.00	
Housemaid drinks alcohol	Yes	20	22	2.71(1.36, 5.39)	0.49(0.18, 1.33)	0.164
	No	50	123	1.00	1.00	
Housemaid uses *shisha*	Yes	21	10	5.47(2.41, 12.42)	2.70(0.94, 7.71)	0.064
	No	51	133	1.00	1.00	
Gender of employer	Male	36	31	2.81(1.48, 5.31)	**2.39(1.05, 5.45)** ^a^	**0.037**
	Female	5	37	0.33(0.11, 0.91)	0.53(0.17, 1.67)	0.284
	Both male and female	31	75	1.00	1.00	
Does your employer chew *chat*?	Yes	55	57	4.88(2.57, 9.24)	**3.78(1.73, 8.29)** ^a^	**0.001**
	No	17	86	1.00	1.00	
Does your employer drink alcohol?	Yes	38	25	5.27(2.90, 9.93)	**2.90(1.17**, **7.17)** ^a^	**0.021**
	No	34	118	1.00	1.00	
Current marital status of housemaid	Single	52	118	0.55(0.28, 1.08)	1.25(0.50, 3.10)	0.628
	Married	20	25	1.00	1.00	

^a^significant variables, *AOR *Adjusted odds ratio, *COR *Crude odds ratio, *CI *Confidence Interval

### Experiences of sexual violence

The prevalence of reported sexual violence among housemaids was 21.4% with 95% CI (15.87, 26.92). The great majority (18.6%) of them reported that they had experienced sexual violence during the one year since the lockdown for the COVID-19 pandemic. About one-third 69 (32.09%) of housemaids experienced unwanted touching and of the 52 housemaids who reported being raped, 21 (40.38%) reported the rape to a friend and 28 (53.84%) to nobody (Table 7).

**Table 7:** Experiences and impacts of sexual violence against housemaids in Kombolcha Town, Ethiopia, January to March, 2021 (*N* = 215)

Variable	Category	Frequency (n)	Percentage (%)
Have you experienced sexual violence?^a^	Yes	46	21.40
	No	169	78.60
Have you experienced sexual violence since COVID-19 lockdown? (*n* = 215)	Yes	40	18.60
	No	175	81.40
Were you raped? (*n* = 215)	Yes	52	24.19
	No	163	75.81
Did someone try to rape you? (*n* = 215)	Yes	55	25.58
	No	160	74.42
Have you experienced unwanted touching? (*n* = 215)	Yes	69	32.09
	No	146	67.91
Rape deal with (*n* = 52)	To nobody	28	53.84
	To a friend	21	40.38
	To a sister/brother	3	5.78
Attempted rape deal with (*n* = 55)	Nobody	25	45.45
	To a friend	24	43.64
	To a sister sister/brother	6	10.91
Did you report being raped? (*n* = 52)	Did not report	45	86.54
	To the *kebele*	3	5.77
	To elders	4	7.69
Did you report attempted rape? (*n* = 55)	Did not report	53	96.36
	To older people	2	3.64
Did you report unwanted touching? (*n* = 69)	Did not report	65	94.20
	To older people	4	5.80

^a^The frequency of sexual violence is smaller than those of rape, attempted rape and unwanted touching because we considered sexual violence to be a composite of rape, making sexually explicit remarks about sex and unwanted touching of the breasts or genital area

The prevalence of reported sexual violence among housemaids was 21.4% with 95% CI (15.87, 26.92). The great majority (18.6%) of them reported that they had experienced sexual violence during the one year since the lockdown for the COVID-19 pandemic. About one-third 69 (32.09%) of housemaids experienced unwanted touching and of the 52 housemaids who reported being raped, 21 (40.38%) reported the rape to a friend and 28 (53.84%) to nobody (Table 7)

### Factors in sexual violence among housemaids

In bivariate logistic regression analysis, age, religion, monthly salary, previous residence and use of alcohol by housemaids and education status, occupation, gender, and use of *chat* by the employer were significant variables at *P*-value < 0.25 and entered into the final multivariable regression model. After controlling for confounding, employers’ alcohol and *chat* use and gender were statistically significant factors of sexual violence in the final multivariate regression model. Housemaids who had male employers were 3.23 times (AOR = 3.23, 95% CI: 1.22–8.52) more likely to have experienced sexual violence than those who had both male and female employers.

Housemaids who had employers who consumed any type of alcohol were 9.72 times (AOR = 9.72, 95% CI: 3.12–20.31) more likely to have experienced sexual violence than their counterparts whose employers did not drink alcohol. Similarly, housemaids whose employer chewed *chat* were 7.40 times (AOR = 7.40, 95% CI: 2.26, 14.21) more likely to have experienced sexual violence than their counterparts whose employers were not *chat* chewers (Table 8).

**Table 8:** Factors associated with sexual violence among housemaids in Kombolcha Town, Ethiopia, January to March 2021 ( *N* = 215)

Variable	Category	Sexual violence		COR (95%CI)	AOR (95%CI)	*p*-value
		Yes	No			
Age of housemaid (years)	14–18	9	57	1.00	1.00	
	19–23	27	82	2.08(0.91, 4.76)	0.81 (0.25, 2.54)	0.718
	24–35	10	30	2.11(0.77, 5.75)	0.85(0.22, 3.23)	0.815
Religion	Christian	36	85	1.00	1.00	
	Muslim	10	84	0.28(0.13, 0.60)	0.89(0.29, 2.69)	0.843
Monthly salary (Birr )	< 978.28	12	71	1.00	1.00	
	≥ 978.28	34	98	2.05 (0.99, 4.24)	1.38(0.51, 3.72)	0.520
Previous residence	Rural	29	75	2.13(1.09, 4.18)	0.62(0.22, 1.72)	0.367
	Urban	17	94	1.00	1.00	
Do you use *shisha*?	Yes	17	14	6.49(2.88, 14.6)	2.63(0.82, 8.43)	0.102
	No	29	155	1.00	1.00	
Employer’s education	Primary school	4	14	1.00	1.00	
	Secondary school	9	36	0.87(0.23, 3.30)	0.36(0.06, 2.15)	0.264
	Diploma or above	29	72	1.4(0.42, 4.64)	0.72(0.12, 4.10)	0.720
	I don’t know	4	47	0.29(0.06, 1.34)	0.14(0.02, 1.03)	0.055
Gender of employer	Male	29	38	5.01(2.38,10.52)	**3.23(1.22**, **8.52)** ^a^	**0.017**
	Female	3	39	0.50(0.13, 1.85)	0.63(0.10, 3.75)	0.616
	Both male and female	14	92	1.00	1.00	
Employer’s occupation	Merchant	9	43	1.00	1.00	
	Government employee	31	77	1.92(0.83, 4.41)	1.76(0.47,4.19)	0.397
	Private worker	6	49	0.32(0.06, 1.63)	4.18(0.83,10.34)	0.080
Does your employer chew *chat*?	Yes	39	73	7.32(3.09,10.31)	**7.40(2.26,14.21**)^a^	**0.001**
	No	7	96	1.00	1.00	
Does your employer use alcohol?	Yes	32	31	10.17(4.85,17.30)	**9.72(3.12, 20.31)** ^a^	**0.001**
	No	14	138	1.00	1.00	

^a^significant variables, *AOR *Adjusted odds ratio, *COR *Crude odds ratio, *CI *Confidence Interval

## Discussion

The current study aimed to assess the prevalence of physical and sexual violence and risk factors on housemaids in Kombolcha Town during the COVID-19 pandemic. The study found one in every three (33.5%) housemaids had experienced physical violence. This finding was consistent with results from a WHO report [5], and studies in Debre Tabor Town [29], other Ethiopian towns [2, 30], and China [31]. But these rates are lower than those reported by another study in Ethiopia [32, 33].

The prevalence of physical violence in our study is higher than found in studies conducted in Mekele [22], Axum [34, 35] and Gondar [36] towns in northern Ethiopia. It is also higher than the reports from UK [37] and Germany [38]. This discrepancy might be due to variations in legal protection of workers, and variations in the time of the studies (the current study was conducted during the early phase of the COVID-19 pandemic). The higher prevalence in our study than in industrialized countries may also be due to the marginalization of Ethiopian housemaids, who tend to lack strong social support and cannot easily protect themselves when living with their employers.

In the current study, about one in five housemaids reported sexual violence. This figure is comparable with several other studies conducted in Ethiopia [25, 39–41], but is higher than in others [30, 42, 43]. The rate in this study was also lower than found in several other studies [44–48]. These variations might be due to contextual factors such as socio-demographic and cultural differences, and the time of study [30]. In the current study gender of employer, and employer and use of alcohol and *chat* by employers were significantly associated with sexual violence. The finding that physical violence was higher among housemaids aged 19–23 years than those 14–18 years old is consistent with previous studies [34, 35, 49–52]. This pattern was associated with physical and mental factors that rendered young housemaids highly vulnerable, which corroborates studies showing that young females were the age group subjected most to sexual violence in other domestic and workplace settings [50, 51].

A study in Nigeria found that underage housemaids were physically abused more than their adolescent counterparts [52]. In addition, perpetrators find it easier to manipulate younger housemaids, further increasing their exposure to violence [30]. The strong correlation between employers chewing *chat* and physical violence is consistent with previous studies [29, 30, 52–58]. *Chat* use reportedly operates as a driver of violence through several pathways. First, physical violence occurs directly through increased anger and aggression brought on by periods of excessive use and withdrawal of *chat*.

Violence may also be facilitated indirectly through multi-step pathways related to the financial and economic burden of *chat* use and its impact on livelihoods [53, 58]. This scenario highlights the importance of addressing substance control, including harm reduction, as part of housemaid violence prevention programs. Housemaids who had employers who consumed alcohol experienced physical violence more often than their counterparts with non-drinking employers. This finding corroborates a study conducted in northwest Ethiopia [29]. Numerous other studies reported that employer alcohol consumption increases the probability of violence [22, 24, 25, 29, 30, 37, 38, 59–64]. Alcohol use may lead to alcohol-induced depressive disorders which can increase the likelihood of employers committing physical violence against their housemaids [22].

The relationship between alcohol and domestic abuse is complicated as alcohol use can have various effects on the perpetrators (employers). These can be situational factors central to the assault itself, such as cognitive impairments caused by alcohol, or more distant but equally important factors, such as negative stereotypes or expectations about sex [65]. The psychopharmacological effect of alcohol on cognitive functioning can cause drinkers to disregard the sexual values and preferences of their victims, a potential key factor in sexual violence [65]. Combined with lowered inhibitions, this can lead to aggression when expectations are not met [66].

According to this study, housemaids who had employers chewing *chat* were more likely to have experienced sexual violence than their counterparts. This finding is consistent with other studies that indicate a positive association between c*hat* chewing and sexual violence [24, 52, 56, 67]. A pathway through which *chat* use leads to violence is elevated and excessive sexual desire, which when coupled with aggressive behavior, leads to sexual violence [66].

Housemaids who had a male employer were more likely to experience sexual violence than housemaids who had both male and female employers, an association not previously reported in the literature. Further studies are needed to assess the nature and extent of gender-based violence in the domestic sphere. Housemaids who had employers who consumed alcohol were more likely to have experienced sexual violence than their counterparts whose employers did not drink alcohol. This finding is consistent with previous studies in various Ethiopia communities [24, 30, 39, 47, 67].

In this study, most housemaids who had experienced forced sex did not report this to a legal body. The main reasons for not reporting to a legal body were lack of knowledge of what to do, cultural taboos, fear of public reaction, shame, and fear of the attacker, exacerbating vulnerability to violence [24, 26]. This is consistent with other studies which demonstrate that violence is a common problem in Ethiopian families, where male domination and female subordination are the cultural norm, curtailing victims’ recourse to social support and legal action [29, 68, 69].

### Strength and limitations of the study

The major strength of this study is that it is the first examination of violence against housemaids in Ethiopia during the COVID-19 pandemic, which may serve as a benchmark for future research. Several limitations should be noted. Since this was a cross-sectional study, participants were assessed only once; thus, it would be difficult to infer the temporal association between a risk factor and outcomes. Further, the cross-sectional nature of the study design cannot show causality between variables. In addition, our definition of sexual violence as a composite of rape and attempted rape, unwanted touching apparently resulted in underreporting of sexual violation. There may be also a self-reporting bias due to sensitive issue of violence and living as a servant might hinders the confidence. The prevalence of sexual violence as well as physical violence, may also have been under-reported due to the sensitivity of this subject and the location of the interviews in the residences of their employers, even though efforts were made to interview participants in privacy.

### Implications of the study

The findings of this study may be useful to assist in the design of appropriate policies addressing the vulnerabilities of housemaids to sexual and physical violence. This study may also provide useful information to labor and social affairs officials and health professionals when planning and implementing violence prevention programs. The high vulnerability of younger housemaids to violence has important implications due to the increasing number of younger girls who are becoming housemaids.

This study may help to design intervention programs that create awareness in the public domain about the many factors related to violence and to ensure that younger housemaids in particular are considered in these programs. This study points out the need for concerned bodies, including police officers, experts in women’s affairs, prosecutors and judges to be equipped with adequate knowledge and skills necessary to effectively and equitably address violence against housemaids. Housemaid protection and empowerment may also be achieved through education, increase in respect for women’s rights, and assistance from women associations at the *kebele* level.

## Conclusion

This study indicates that more than one-third of the housemaids and nearly one in every five housemaids experienced physical and sexual violence, respectively. Housemaids in Kombolcha Town have experienced more physical and sexual violence during the COVID-19 pandemic. The most vulnerable housemaids were in the youngest age group of 19–23 years and had a male employer, particularly an employer who used alcohol or *chat.* The findings point out an urgent need for an integrated policy that addresses the rise of physical and sexual violence against housemaids by their employers. Enhancing awareness on the role of *chat* chewing and alcohol use in violence should be an integral component of violence reduction programs. In addition, targeted efforts should be made to reduce the vulnerability of younger housemaids.

### Supplementary Information

**Additional file 1.**

## Data Availability

The data used for analysis is fully available in the manuscript file without restriction.

## Abbreviations

AOR: Adjusted Odds Ratio
CI: Confidence Interval
COR: Crude Odds Ratio
STI: Sexually transmitted infections
WHO: World Health Organization
