# Supplementary material for: Violence against housemaids in an Ethiopian town during the early phase of the COVID-19 pandemic: a cross-sectional study
Source: BMC Womens Health. 2023 Sep 12;23:485. doi: 10.1186/s12905-023-02530-w (PMC10498593; doi:10.1186/s12905-023-02530-w)
Supplement: Supplementary file 1 — Additional file 1. [file 12905_2023_2530_MOESM1_ESM.docx]

Additional file1

Data collection Tool

Name of interviewer__________________________ Date of interview________________________

Kebele____________________________ House number_______________________________

**Section 1: Socio-demographic and other characteristics of respondents**

| No | | Questions | | Coding category |
| --- | --- | --- | --- | --- |
| 101 | | What is your age? | | ----------years |
| 102 | | What is your religion? | | 1. Orthodox  2. Catholic  3.Protestant 4. Muslim |
| 103 | | Marital status? | | 1. Never married 2. Married 3. Living with a partner or boyfriend 4. Divorced 5. Widowed |
| 104 | | Educational status | | 1.Unable to read and write  2. No formal education 3. Primary education  4. Secondary education  5.University student |
| 105 | | What is Your monthly income? | | ---------------in Birr |
| 106 | | Where did you grow up? | | 1. Rural 2. Urban |
| 108 | | How long have you worked for your current employers | | 1) < a year, 2) 1-3 years, 3) > 3 years |
| 109 | | Type agreement of employment with the employer | | 1. Written contract 2. Daily 3. Permanent |
| \| 110 \| \| --- \| | | With whom are you living? | | 1. With my employer 2. With my family 3. With Husband/Boyfriend 4. With female friends 5. Alone |
| 113 | | Can you tell me why you choose to work as a housemaid? | | 1. Lack of other employment opportunities 2. My parents divorced 3. Self-divorce (employee) 4. Death of parents 5. Opposing early marriage 6. other_____________ |
| **Employees Family history** | | | | |
| 201 | Are your father and mother currently living together? | | 1. Yes 2**.** No, divorced   3. Only Mother alive  4. Only Father alive  5. Neither of them is alive | |
| 202 | Father’s educational status | | 1. Illiterate  2.Readandwrite  3. Primary school  4. secondary school  5. Higher education | |
| 203 | Mother’s educational status | | 1. Illiterate  2.Readandwrite 3. Primary school  4.Secondaryschool 5. Higher education | |
| 204 | Do you think that you are receiving enough money for your work as a housemaid? | | 1. Yes 2. No | |

# Part two: Behavior-related questions about Housemaids

| 301 | Do You drink alcohol? | 1.Yes 2. No (skip to 303) |
| --- | --- | --- |
| 302 | If so how often do you drink? | 1. Regularly 2. Sometimes |
| 303 | Do you chew chat? | 1. Yes 2. No(skip to Q305) |
| 304 | If yes how often? | 1. Regularly 2. Sometimes |
| 305 | Do you take the following drugs: 1.cocaine 2.shisha 3.marijuana | Yes No 1 2 1 2 1 2 |

**Employers’ characteristics**

| 401 | Employers education level | 1. Illiterate 2. Primary school 3. Secondary school 4. Diploma and above 5. I don’t know |
| --- | --- | --- |
| 402 | What is the gender of your employer? | 1) male 2) female 3) both male and female |
| 404 | Employer’s occupation | 1. Merchant/shop owner 2. Government employee 3. Private worker 4. I don’t know 5. Other specify |
| 406 | Have your employers ever chewed chat since you join this home? | 1. yes 2. NO (skip to Q408) |
| 407 | How often does your employer chew chat? | 1. Every day 2. Once or twice a week   3. 1 -3 times a month 4. Occasionally, less than once a month |
| 408 | How often does your employer used chat since you started working in this home? | 1. Every day or nearly every day  2. Once or twice a week 3. 1 – 3 times a month 4. Occasionally, > once a month |
| 409 | Did your current employer ever consume alcohol (beer, talla, tej, wine, whiskey, gin etc.) since you worked for him/her? | 1. Yes 2. No |
| 410 | How often does your current employer drink alcohol since you worked for him/her? | 1. Every day or nearly every day 2. Once or twice a week 3. 1-3 times a month 4. Occasionally, (< once a month) |
